# Gruberi bursitis in rheumatic patients with foot and ankle pain: a retrospective sonographic study

**DOI:** 10.3389/fmed.2025.1501468

**Published:** 2025-02-14

**Authors:** Plamen Todorov, Lili Mekenyan, Boryana Levterova, Anastas Batalov

**Affiliations:** ^1^Department of Internal Disease Propaedeutics, Medical University of Plovdiv, Plovdiv, Bulgaria; ^2^Clinic of Rheumatology, Kaspela University Hospital, Plovdiv, Bulgaria; ^3^Department of Health Management and Healthcare Economics, Faculty of Public Health, Medical University of Plovdiv, Plovdiv, Bulgaria

**Keywords:** musculoskeletal ultrasound, rheumatic, foot, ankle, pain, Bursa

## Abstract

**Introduction:**

Foot and ankle pain is a common problem in rheumatic patients. One often underrecognized cause of this complaint is Gruberi (or subtalar) bursitis. The Gruberi bursa is a structure that originates from the sinus tarsi and extends over the dorsal talar surface. It reduces the friction of the extensor digitorum longus tendon over the convex contour of the head of the talus. On ultrasound, Gruberi bursitis is characterized by a well-defined fluid collection in the dorsolateral foot, located between the talus and the tendon of the extensor digitorum longus. Our study aimed to determine the frequency of Gruberi bursitis in patients with various rheumatic diseases who presented with foot and ankle pain at our institution and to describe its sonographic features in detail.

**Materials and methods:**

A descriptive, observational, retrospective study was conducted on patients over 18 years old who visited a tertiary university hospital between 1 July 2022 and 31 December 2023. Details regarding the patients’ medical history, age, gender, and primary rheumatic disease were obtained from their medical records. Descriptive statistics were utilized to present the data.

**Results:**

Of the 608 patients examined for foot and ankle pain at our institution during the study period, 78 cases of Gruberi bursitis were identified in 63 patients. The average age of the participants was 61.7 years (range: 25–85 years), and 71% (*n* = 45) of the participants were women. The sonographic features of Gruberi bursitis included a monocular, anechoic fluid collection typically located between the extensor digitorum longus tendon and the dorsolateral surface of the head of the talus. The mean largest dimension of fluid collections in the oblique plane (from the sinus tarsi and across the dorsal talus) was 16 mm (range: 8–29 mm).

**Conclusion:**

Gruberi bursitis is easily identifiable through ultrasound due to its characteristic location and appearance. In total, we identified this condition in 9% of our rheumatic patients with foot and ankle pain.

## Introduction

Foot and ankle pain is a common problem in rheumatic patients. It could originate from various structures within the foot itself or be referred from more cranial sites (1). Musculoskeletal ultrasound (MSUS) has increasingly been used as a first-line imaging method in rheumatology. It allows for the easy identification of abnormal fluid collections and inflammatory, degenerative, or traumatic pathologies in various structures within this anatomical region (2). Bursae are sac-like structures containing a small amount of synovial fluid that reduce the friction between soft tissues and bones during motion (3). Bursitis is a common condition characterized by irritation, inflammation, and swelling of the bursa capsule and its contents, which can elicit pain and reduce the normal range of motion (4). Normal bursae may not always be seen using MSUS or they may appear as structures with thin hyperechoic walls and small anechoic, film-like content (5). Inflamed bursae are generally present with an increased amount of synovial fluid (of variable echogenicity), with or without synovial hypertrophy, internal septation, mural nodules, or loose bodies. However, no specific appearance can typically be linked to any particular etiology (6). Recent advancements in sonographic research have divided the ultrasound presentation of bursitis into exudative and hypertrophic patterns. The exudative pattern is characterized by a thin synovial lining coupled with varying amounts of anechoic effusion inside the bursal cavity. The hypertrophic pattern presented substantial thickening of the intimal and subintimal layers of the bursal synovium, which can appear in different morphological forms, including linear, villous, or pseudotumoral patterns (7). Bursae around the foot and ankle can be categorized as anatomic and adventitious. Anatomical bursae are present at birth in established anatomic locations, while adventitious bursae develop later at sites of increased or repetitive friction (8). An anatomic bursa found in the dorsolateral ankle is termed the sinus tarsi or Gruberi bursa (GB) and was first described by Alexander Monro in 1825 (9). This bursa originates from the sinus tarsi, extending cranially at first and then medially above the talus to occupy the space between the extensor digitorum longus tendon (EDLT) and the dorsolateral talar bone surface (10). Since its description in the 19th century, this anatomical structure has received little attention, and there is limited data about it in the literature. On the other hand, in our clinical practice, when assessing the feet of rheumatic patients using MSUS, we frequently encountered a hypoechoic, compressible cyst-like structure in the dorsolateral foot. As this corresponds to the description of the GB provided by various authors, we decided to perform a retrospective analysis of our center’s database regarding the frequency and characteristics of this finding. Our ultrasound database was searched for studies performed over an 18-month period, from 1 July 2022 to 31 December 2023, to identify participants with dorsolateral foot fluid collection detected by MSUS scanning. Images were extracted and retrospectively reviewed to characterize the location and size of the fluid collection and describe the sonographic features of GB in our patients.

### Aim

This study aimed to determine the frequency of GB in various rheumatic diseases treated at our center and to describe its sonographic features in detail.

### Materials and methods

A descriptive, observational, retrospective study was conducted on patients over 18 years old who visited the Rheumatology Clinic of University Multiprofile Hospital for Active Treatment (UMHAT) Kaspela Ltd., Plovdiv, Bulgaria, from 1 July 2022 to 31 December 2023. The inclusion criteria were as follows: (1) patients over 18 years old; (2) established rheumatic disease; and (3) the presence of unilateral or bilateral foot and/or ankle pain in patients, regardless of their underlying rheumatic disease, who had undergone ultrasound evaluation of the foot and/or ankle regions due to this complaint.

We searched our department’s ultrasound database to identify reports describing fluid collections or bursae in the dorsal foot area of these patients. We used the following terms “GB,” “subtalar bursa,” “sinus tarsi,” “bursitis,” and “tenosynovitis” to search our database and included MSUS scans of the feet and ankles obtained between 1 July 2022 and 31 December 2023. Reports that contained any of these terms were identified, and the corresponding MSUS exams were extracted and analyzed for the presence of a well-defined fluid collection beneath the EDLT and above the dorsolateral talus, originating from the sinus tarsi. Images that did not have adequate quality of the assessed structures in both longitudinal and transverse planes were excluded.

**Figure 1:**
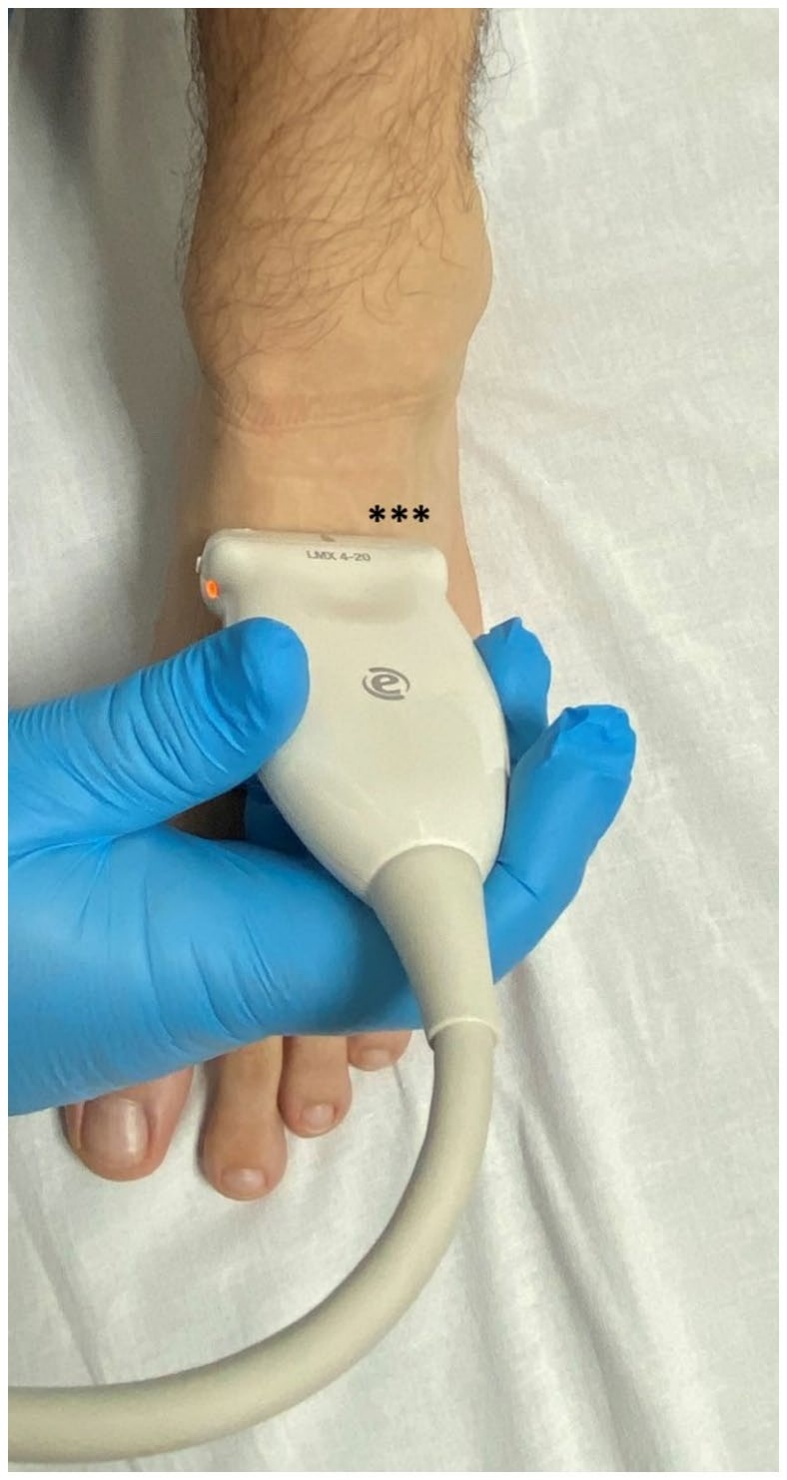
Position of the transducer over the dorsal foot in the short axis. The location of the GB is in the dorsolateral part of the foot (marked by *).

Ultrasound imaging was performed by a rheumatologist with 10 years of experience in MSUS, using two ultrasound machines (Logic E9, GE Healthcare, USA, and Esaote My Lab X8, Genoa, Italy). Both machines were equipped with multifrequency linear transducers: ML 6–15 and LX 3–15 MHz, respectively. Ultrasound examination of the foot and ankle is part of the comprehensive evaluation of patients with various rheumatic diseases who present with complaints in these anatomical areas. The standard MSUS protocol used in our department follows the EULAR recommendations for the ultrasound assessment of the foot and ankle (Figure 1) (11). Power Doppler (PD) was used as a supplementary method after greyscale (GS) scanning, with a pulse repetition frequency (PRF) of 0.7, a wall filter setting of 4, and a frequency of 10 MHz. The clinical examination reports of the patients with MSUS evidence of the GB were also reviewed to determine whether they presented with focal warming, pain, or swelling and to record their primary rheumatological diagnosis.

### Image review process

An imaging review was performed by consensus between two rheumatologists trained in MSUS, one with more than 10 years of experience and the other with 3 years of experience. This review aimed to define the presence and characteristics of the GB. The following imaging data were recorded for each case of confirmed bursitis: presence of fluid (anechoic or hypoechoic), location of fluid (i.e., the location of the epicenter of the collection), size of the fluid collection (largest dimension in millimeters), number of bursal locules (unilocular or multilocular), internal content of the collection, and presence of a PD signal. When bilateral collections were recorded, they were assessed and described separately.

### Clinical data

Clinical data were reviewed from the electronic medical records at our institution to collect information regarding the patients’ medical history, age, gender, and primary rheumatological diagnosis, as well as their main complaints at the time of the MSUS examination.

### Statistical analysis

Statistical analysis was conducted using IBM SPSS Statistics, version 26 (IBM Corp, USA). Descriptive statistics were utilized to present the data. The sample data were reported as frequencies (N) and percentages (%) for categorical variables and as mean values and standard deviations (SDs) for continuous variables. A chi-squared test was performed to assess the association between the categorical variables, and the independent-sample Mann–Whitney U test was performed to compare the continuous variables. A *p*-value of <0.05 (two-tailed) was considered statistically significant.

## Results

### Ultrasound image review

**Figure 2:**
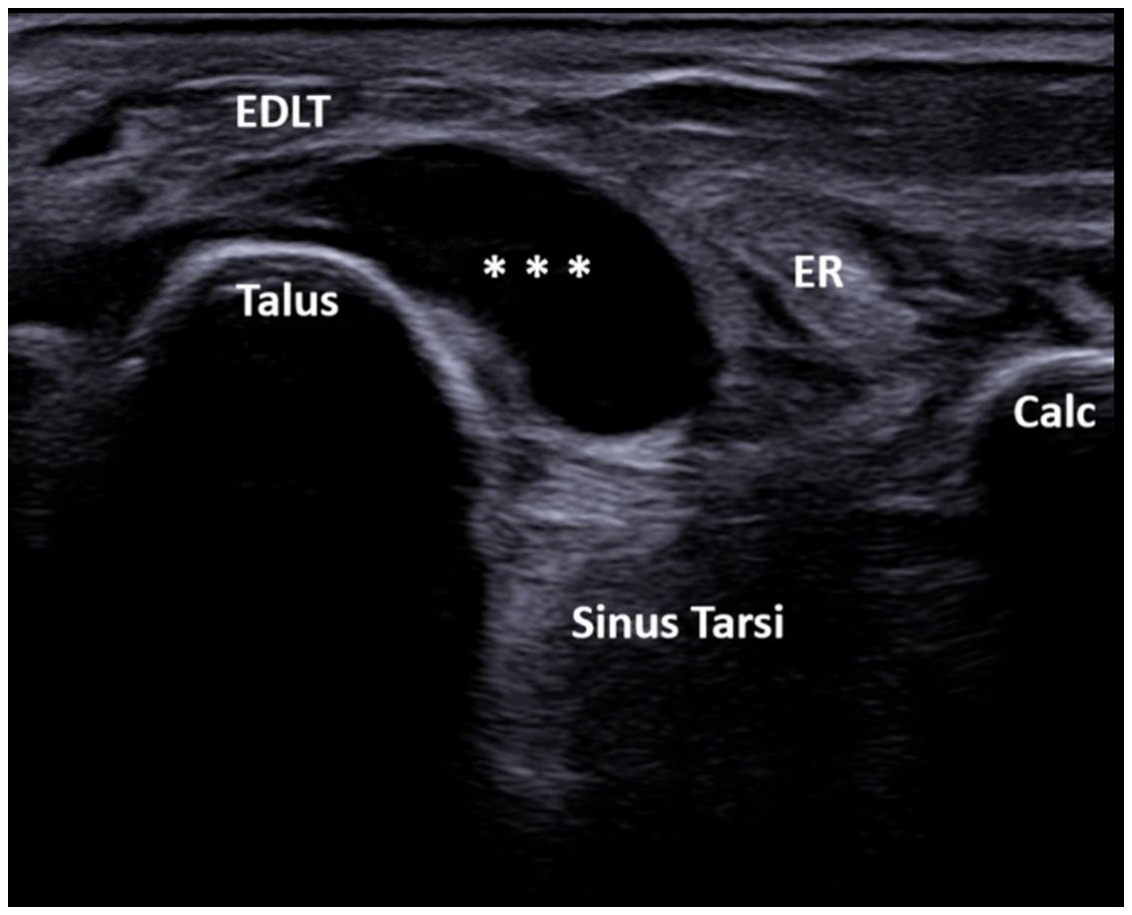
A 60-year-old patient with systemic sclerosis. Ultrasound image showing unilocular GB with the transducer positioned in the short axis of the foot (left side is medial). The transverse ultrasound image of the dorsolateral ankle shows an anechoic unilocular fluid collection (***) between the extensor digitorum longus tendon (EDLT) and the talus. ER, extensor retinaculum; Calc, calcaneus; and EDLT, extensor digitorum longus tendon.

**Figure 3:**
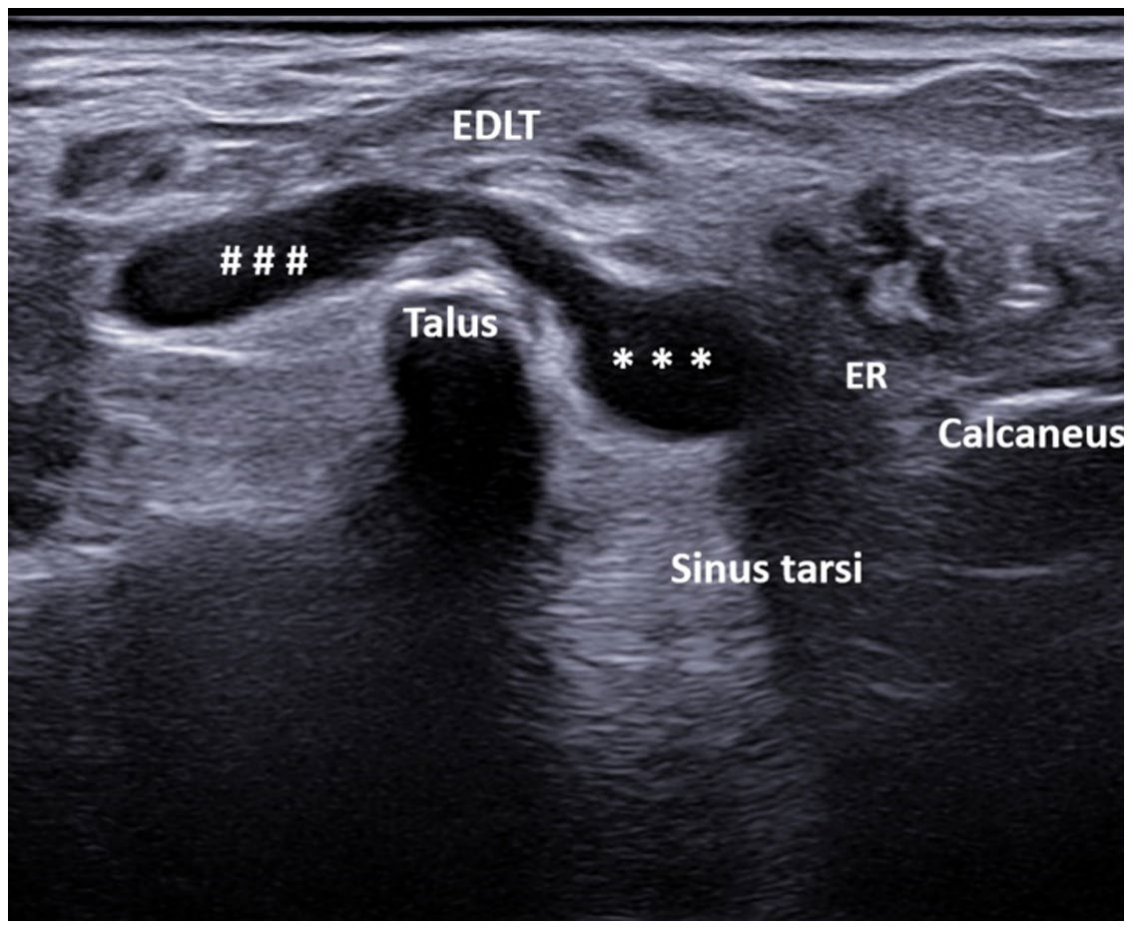
A 44-year-old patient with spondyloarthritis. Ultrasound image showing bilocular GB with the transducer positioned in the short axis of the foot (left side is medial). The transverse ultrasound image of the dorsolateral ankle reveals a bi-lobed, anechoic fluid collection (* – lateral lobe, # – medial lobe) between the extensor digitorum longus tendon (EDLT) and the talus. ER, extensor retinaculum and EDLT, extensor digitorum longus tendon.

**Figure 4:**
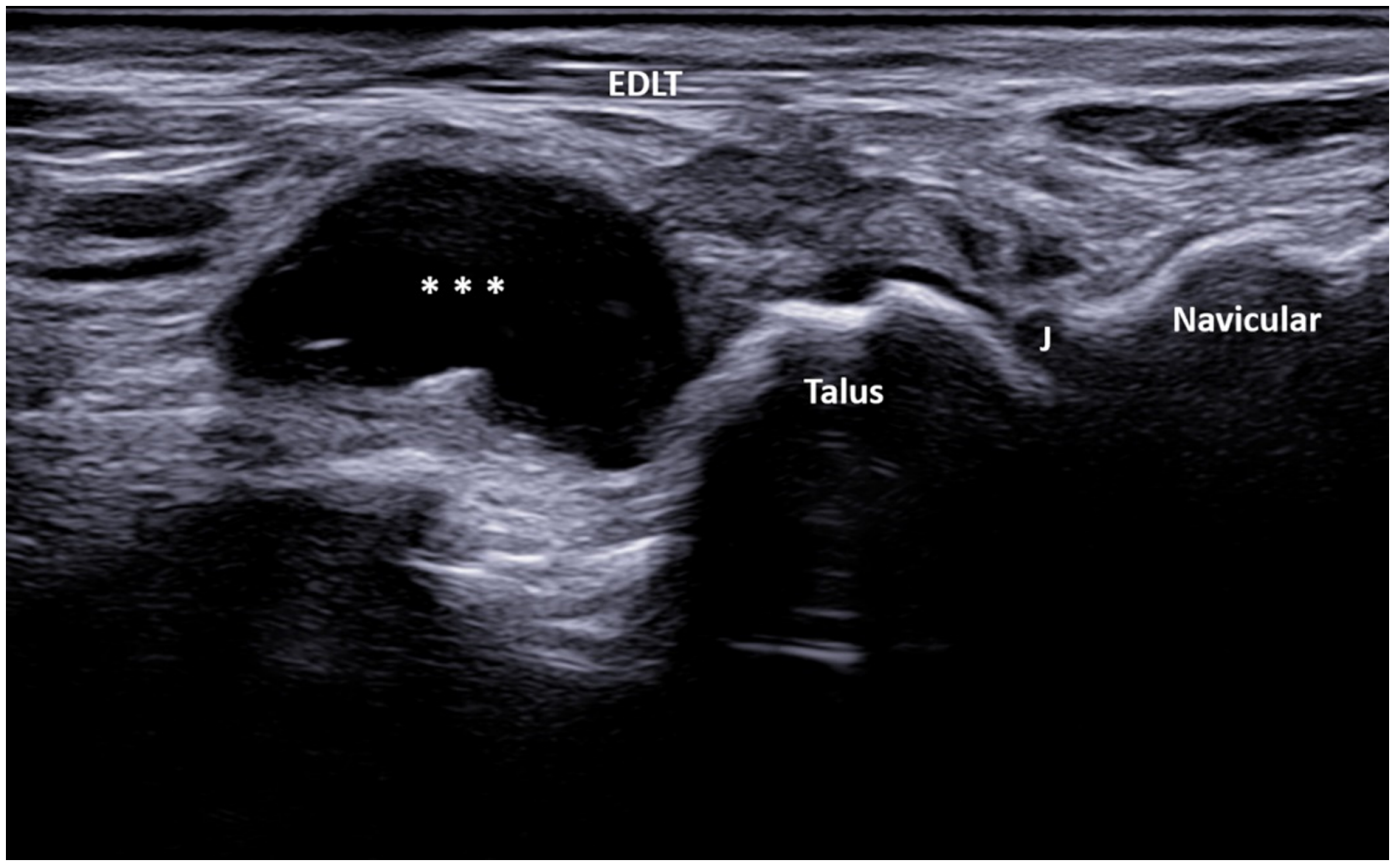
A 67-year-old patient with osteoarthritis. Ultrasound image showing GB with the transducer positioned in the long axis of the foot (left side is proximal). The longitudinal ultrasound image of the dorsolateral ankle reveals an anechoic unilocular fluid collection (***) between the extensor digitorum longus tendon (EDLT) and the talus. J, talonavicular joint and EDLT, extensor digitorum longus tendon.

**Figure 5:**
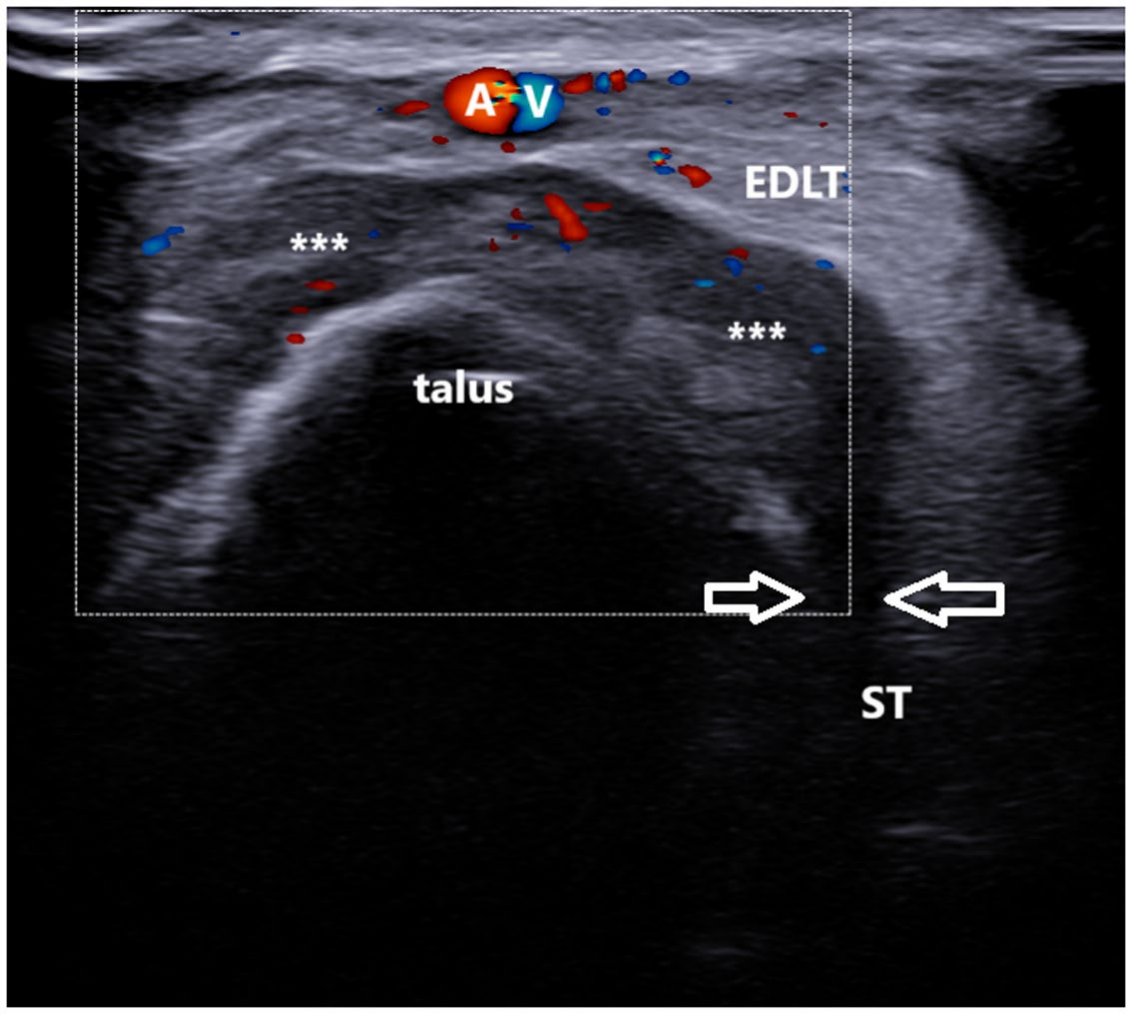
A 37-year-old patient with gout. Ultrasound image showing unilocular, power Doppler-positive GB with the transducer positioned in the short axis of the foot (left side is medial). The transverse ultrasound image of the dorsolateral ankle reveals a hypoechoic to heteroechoic unilocular fluid collection (***) between the extensor digitorum longus tendon (EDLT) and the talus, extending from the sinus tarsi (between arrows). ST, sinus tarsi; A and V, dorsalis pedis artery and vein; and EDLT, extensor digitorum longus tendon.

A total of 608 patients underwent ultrasound examination for foot and/or ankle pain in our department from 1 July 2022 to 31 December 2023. Of these 608 patients, the search based on the previously cited keywords identified 76 patients who met the inclusion criteria. After reviewing the images of these patients, 13 were excluded for the following reasons: fluid collection was seen only deep within the sinus tarsi without spreading above the dorsolateral talar contour and beneath the EDLT (3 patients), tenosynovitis of the EDLT was diagnosed (6 patients), or no fluid was actually seen (4 patients). Thus, the final study group consisted of 63 patients with different primary rheumatic diagnoses (Table 1). Of these, the finding was unilateral in 48 patients and bilateral in 15 patients, resulting in a total of 78 bursae analyzed. Of the 63 patients, 45 (71%) were women and 18 (29%) were men. The average age of the patients was 61.7 years (range: 25–85 years). The right foot was affected in 55% (44/78) of the cases, and the left foot was affected in 45% (34/78) of the cases. The fluid collection extended from the sinus tarsi, along and beneath the inferior extensor retinaculum, reaching the space between the EDLT and the dorsolateral surface of the head of the talus. The large bursae also extended medially from this space. The bursae were predominantly unilocular (49/78) rather than multilocular (29/78). In all cases, the epicenter of the fluid collection was situated in the plane between the EDLT and the dorsolateral talar contour (Figure 2, Supplementary Video 1). When present, the second loculus was located medially to the main loculus and positioned over the talus (Figure 3). The majority of the fluid collections were clearly anechoic (69/78) (Figures 2, 4). In the remaining cases, internal hyperechoic foci were observed (Figure 5), making the content heteroechoic, and an internal septum was present in one case.

**Table 1:** Primary rheumatic diagnoses in our patients with GB (*N* = 63).

Rheumatoid arthritis	20
Osteoarthritis	19
Spondyloarthritis	11
Cristal arthropathies	8
Systemic sclerosis	3
Lupus Erythematosus	1
Polymyalgia rheumatica	1

In the longitudinal plane, the GB had an ellipsoid shape and was located between the EDL tendon and the talus (Figure 4). Its middle portion was the thickest, while the inferior and superior poles narrowed sharply. We did not notice synovial hypertrophy in any recorded bursae. PD signals were registered in only four cases (Figure 5, Supplementary Video 2). Due to the small number of the PD-positive bursitis cases, the exact pattern (i.e., internal or along the bursal wall) and grade of the signal were not analyzed separately.

The mean largest dimension of the fluid collections between the EDLT and the talus was 10 mm (range: 2–23 mm). The mean largest dimension of the fluid collections in the oblique plane (from the sinus tarsi and across the dorsal talus) was 16 mm (range: 8–29 mm). In 59 cases (76%), sonopalpation of the fluid collection was recorded as positive, meaning the patients reported pain when pressure was applied with the transducer over the identified fluid collection.

In three patients, local swelling over the dorsal foot was observed, while in two patients, overlaying erythema was recorded in the clinical records. The GB was painful enough to necessitate aspiration and steroid injections in only five patients. The microscopic examination of the fluid revealed uric acid crystals in two patients, calcium pyrophosphate crystals in one, and a non-specific inflammatory aspirate in two others.

### Clinical data

The underlying clinical diagnoses of the patients are described in Table 1. All of them were referred for scanning due to complaints of pain and stiffness or decreased range of motion in the ankle and foot. The foot and ankle complaints were not exclusive for the majority of these patients as they were also experiencing symptoms in other anatomical regions. All patients were on various therapies for their primary diagnoses. Thus, an in-depth analysis of the patients’ overall status and the activity or stage of their primary rheumatic disorder at the time of the ultrasound assessment and GB identification was considered not relevant to this study and was not attempted. In addition, no data could be extracted on whether the presence of the GB influenced the physicians’ treatment decisions, except in the five cases where aspirations were performed.

## Discussion

The GB is an anatomical bursa located in the dorsolateral ankle. It has been described previously but has received little attention in the contemporary musculoskeletal or ultrasound literature. This bursa was first described by Alexander Monro (1773–1859) as “a bursa mucosa for the tendons of the extensor digitorum communis longus, between them and the tibia and ligament of the ankle” (9). Several later studies described a bursa between the dorsolateral surface of the talus and the EDLT, with some suggesting a possible communication between the bursa and the talonavicular joint (12–14). However, none reported a communication between the bursa and the tibiotalar joint.

In a recent report, Weerakkody and Murphy (15) defined the GB using magnetic resonance imaging (MRI) as the bursa that lines the inferior extensor retinaculum while it passes over the EDLT at the anterior ankle joint. It is usually only visible on imaging if it is distended and may be mistaken for tenosynovitis, which is why it is also referred to as “pseudo-tenosynovitis” of the EDLT. However, the fluid in the GB extends from the sinus tarsi and wraps around the talus, distinguishing it from the fluid in EDL tenosynovitis, which extends along the tendon.

In our study, the GB was identified using MSUS as a structure distinct from both the tibiotalar and talonavicular joints, extending from the sinus tarsi along the inferior extensor retinaculum to the inferior surface of the EDLT, with varying sizes. The epicenter of the fluid collection, i.e., its thickest part, was always between the EDLT and the dorsolateral talus. In the longitudinal plane, the GB had an ellipsoid shape and was located between the EDL tendon and the talus (Figure 4). Its middle portion was the thickest, while the inferior and superior poles narrowed sharply. The margins of the bursa were well-defined in both the transverse and longitudinal planes, and it was clearly distinguishable from the EDLT, talonavicular joint, and tibiotalar joint.

Anatomic bursae, unlike adventitious ones, are lined with synovial cells, may contain fluid, and sometimes communicate with joints (16). When collapsed, bursae may not be visible on ultrasound, while a bursa distended with fluid typically appears to be anechoic, unilocular, and compressible on ultrasound (5, 6). Some bursae, even when distended, are asymptomatic, whereas others can cause pain, swelling, nerve compression, and even osseous erosion (17). Bursae can be confused with ganglion cysts because of their clear anechoic fluid content and potential communication with joints. However, anatomic bursae typically occur at predictable anatomic locations, which helps distinguish them from ganglion cysts. In addition, ganglion cysts are typically non-compressible on ultrasound and contain more viscous fluid (18).

A recent cadaveric and ultrasound study identified a small (i.e., non-distended), unilocular, compressible fluid collection overlaid by the EDLT, a location corresponding to the known site of the GB (19). Subsequent latex injection and dissection of the specimen, performed by the authors, confirmed that the latex had been deposited within a thin-walled cavity between the EDLT and the talus and that the latex had extended toward the sinus tarsi along the inferior extensor retinaculum. This study thus confirmed the possibility that the GB, when distended, could be well visualized and assessed using MSUS. The same study found that the prevalence of GB was 2.2% among patients referred to a musculoskeletal ultrasound department due to ankle and/or foot pain. Of these patients, only 37% had documented pain when pressure was applied using the transducer (sonopalpation) over the bursa, while 63% tested negative on sonopalpation. However, it should be noted that this maneuver was performed in only a minority of patients (27/177). In addition, 98% of the detected GB were unilocular, and 94% had an anechoic content (19).

Our study reported a slightly different picture: 75% of the GB detected were positive (painful) on sonopalpation (a maneuvre that is routinely performed in our practice for each sonopathological finding), while 37% were multilocular. In four cases (two patients with rheumatoid arthritis (RA) and two patients with crystal arthritis), PD signals were recorded, while Gaetke-Udager et al. did not report such cases (19). In addition, a local warming of the overlaying skin was recorded in two patients from our study. These differences could be explained by the different patient populations. While the study by Gaetke-Udager et al. was set in a musculoskeletal ultrasound unit that receives referrals from different departments, the patients in our study were exclusively referred from rheumatological practices, and the majority (44/63) had inflammatory rheumaticdiseases.

The GB identified in our study was found to be more prevalent among female patients (71% of the patients). One explanation for this discrepancy could be that women tend to wear tighter shoes and high heels, which cause friction of the EDLT along the dorsolateral ankle and the head of the talus, resulting in fluid accumulation in the bursa.

The two rheumatic conditions that accounted for more than half of the GB cases in our study population (39/63) were rheumatoid arthritis (RA) and osteoarthritis (OA) (with 20 and 19 patients, respectively). As anatomic bursae are lined with synovial cells (14), the involvement of these structures is common in RA (20). This may explain the leading prevalence of RA among our patients with GB. In fact, intermetatarsal bursitis has been found to be highly frequent in early RA (21), but there are no data on the GB. OA, on the other hand, is a common disease affecting the ankle and foot joints, though it rarely leads to overt synovial inflammation (22). Nevertheless, it was the second most common rheumatic condition among our patients with GB. There could be at least two explanations for the frequency of GB in OA: firstly, knee and hip OA could cause disturbances in the gait and leg axis, which could lead to increased friction in the dorsolateral foot region (23). However, a dedicated and comprehensive gait analysis is not routinely performed in our department. Secondly, the involvement of the neighboring talonavicular joint by OA could cause proliferative bone changes (osteophytes) that may irritate the bursa, predisposing it to inflammation (24).

Our review of the ultrasound studies shows that the primary feature identifying the GB is its location between the EDLT and the distal dorsal talus, which is consistent with its original anatomic descriptions (10). This bursa is easily visualized using MSUS, as it is superficial, situated over a bony contour, and has a typical shape. It can be clearly differentiated from the neighboring synovium-lined structures, such as the EDLT, the tibiotalar joint, and the talonavicular joint. The pattern of fluid distribution, if these structures are inflamed, is expected to differ—either along the course of the EDLT or with a clear connection to the aforementioned joints’ cavities. Indeed, in the performed initial image analysis, six cases of EDLT tenosynovitis were identified, and these patients were excluded from the study. No connection with the talonavicular or tibiotalar joints was identified in any patient. A recent cadaveric study described in detail the lateral part of the tibiotalar joint, which is anatomically closest to the location of the GB (25). A synovial cyst or dilated recess could generate similar complaints and potentially be mistaken for GB. However, the fluid collection should be traceable to the tibiotalar joint cavity, and dynamic examination with ankle flexion/extension and foot eversion/inversion could further help in differentiating these conditions.

**Figure 6:**
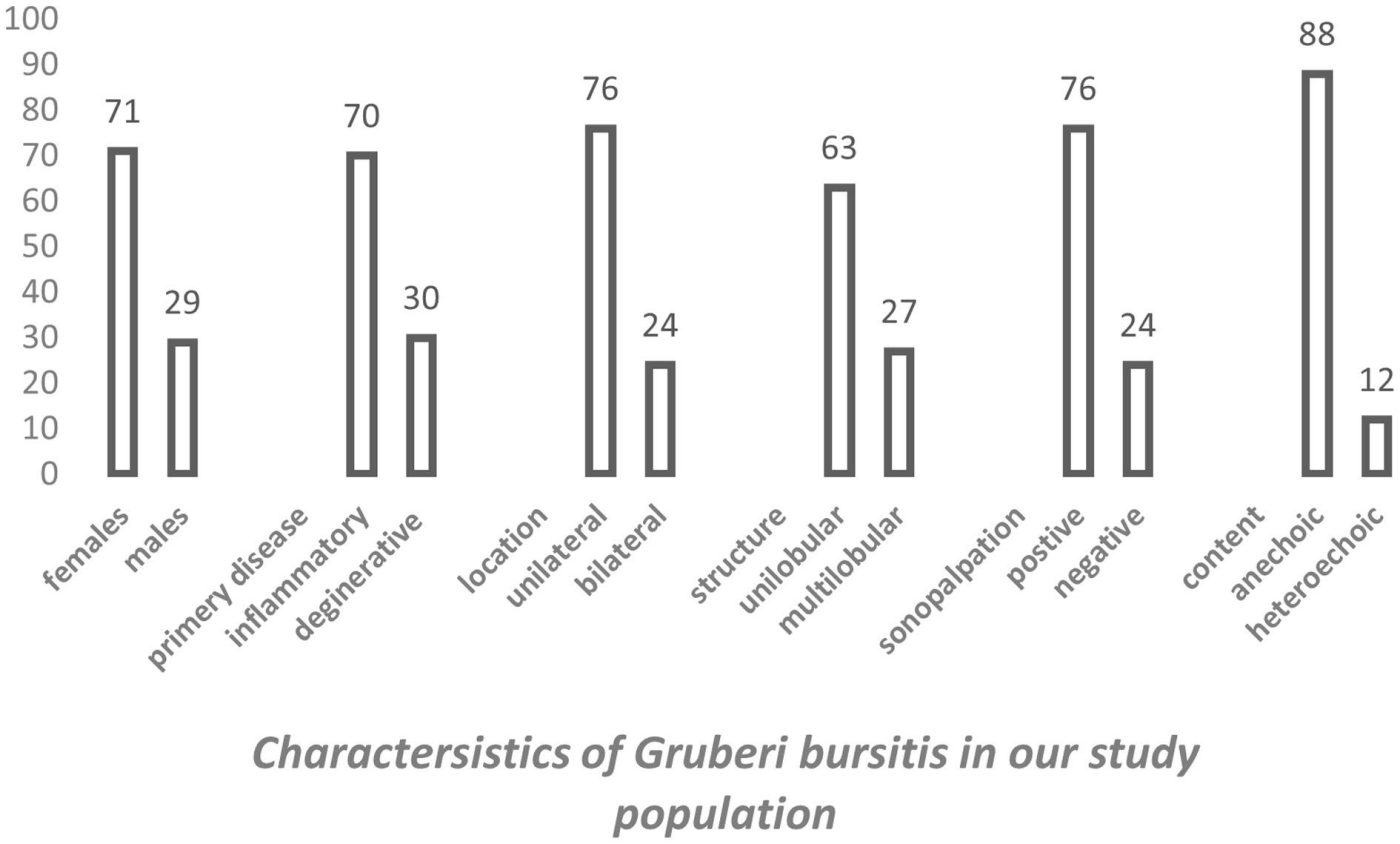
Characteristics of GB in our study population (63 patients, 78 bursae).

We identified GB in 9% of the rheumatic patients with ankle and/or foot pain in our department, and in 7% of these cases, it was painful upon sonopalpation. Of the GB cases included in our retrospective image review, the majority were unilocular (49 vs. 29), anechoic (69 vs. 9), did not exhibit PD (74 vs. 4), were painful on sonopalpation (59 vs. 19), affected only 1 foot (48 vs. 15), and were more frequent in female patients (45 vs. 18) and in the patients with inflammatory rheumatic diseases (44 vs. 19) (Table 2, Figure 6).

**Table 2:** Most common sonographic findings of GB (N 78).

Unilocular	62.8%
Anechoic	88.4%
Compressible	100%
Sonopalpation positive	75.6%
PD negative	94.9%

PD, Power Doppler.

Our study, although retrospective, has certain clinical implications. Firstly, our results showed that GB, contrary to previous publications (10, 15, 19), is not rare in patients with rheumatic disorders who experience pain in the dorsal foot. Thus, this pathology should be considered when examining such patients. Secondly, GB has a characteristic MSUS appearance, allowing for its easy identification and reliable differentiation from the pathology of the neighboring synovial-lined structures. Therefore, assessing for possible GB should be part of the routine ultrasound scanning of the ankle and foot.

Our study has several limitations, the primary one being the lack of surgical proof or MRI confirmation of the presumed GB. However, the cadaver and anatomic descriptions from previous studies, as well as the magnetic resonance imaging data from published case studies, are consistent with our data and clearly indicate that the ultrasound findings are of GB. Another limitation is that the ultrasound examinations were performed by the same rheumatologist; however, this actually ensured the consistency of the scanning method and protocol. In addition, the images were reviewed retrospectively by two other rheumatologists who had to reach a consensus. Lastly, our study, being retrospective, had the internal limitation of using only previously recorded images, which prevented us from comparing the GB with other fluid collections in the ankle and foot that might be symptomatic and could coexist with the GB. In conclusion, the GB is characteristically identified as a fluid collection that extends upwards from the sinus tarsi along the inferior extensor retinaculum to reach the space beneath the EDLT. The thickest part of this fluid collection is located between the tendon and the dorsolateral talar head. On ultrasound, the GB is most commonly unilocular, anechoic, and compressible. One should be aware of the location and the characteristics of this bursa when performing ultrasound examinations of the ankle and foot in patients with rheumatic diseases.

## Data Availability

The original contributions presented in the study are included in the article/Supplementary material, further inquiries can be directed to the corresponding author.
